# Rootstocks Comparison in Grafted Watermelon under Water Deficit: Effects on the Fruit Quality and Yield

**DOI:** 10.3390/plants12030509

**Published:** 2023-01-22

**Authors:** Carolina Morales, Camilo Riveros-Burgos, Felipe Espinoza Seguel, Carlos Maldonado, Jacob Mashilo, Catalina Pinto, Rodrigo Iván Contreras-Soto

**Affiliations:** 1 Institute of Agri-Food, Animal and Environmental Sciences (ICA3), Universidad de O’Higgins, San Fernando 3070000, Chile; 2 Centro de Genómica y Bioinformática, Facultad de Ciencias, Universidad Mayor, Santiago 8580745, Chile; 3 Agriculture Regulatory and Technology Development Directorate, Towoomba Research Centre, Limpopo Department of Agriculture and Rural Development, Private Bag X1615, Bela-Bela 0480, South Africa

**Keywords:** deficit irrigation strategy, water productivity, crop evapotranspiration, root system

## Abstract

Drought is widely recognized as one of the most significant agricultural constraints worldwide. A strategy to avoid the adverse effects of drought on crops is to cultivate high-yielding varieties by grafting them onto drought-tolerant rootstocks with a differentiated root system. Thus, the objective of this study was to evaluate fruit yield and quality, root system architecture, and water productivity of watermelon grafted onto *Lagenaria siceraria* rootstocks. To do so, a commercial watermelon cultivar “Santa Amelia” [*Citrullus lanatus* (Thunb.)] was grafted onto five *L. siceraria* rootstocks: ‘Illapel’, ‘Osorno’, ‘BG-48’, ‘GC’, and ‘Philippines’, which were grown under three irrigation treatments (100%, 75%, and 50% of evapotranspiration). The comparison of the *L. siceraria* rootstocks in the irrigation treatments demonstrated no significant effect on watermelon fruit quality parameters. The rootstocks ‘Illapel’, ‘Osorno’, and ‘GC’ significantly improved the fruit number and yield (total fruit weight) under water deficit. Similarly, ‘Illapel’, ‘Osorno’, and ‘GC’ consistently showed statistical differences for root system architecture traits compared to ‘BG-48’ and ‘Philippines’. Based on these results, we concluded that the used *L. siceraria* rootstocks did not affect the fruit yield and quality of grafted watermelon under water deficit. This study may help adjust the amount of applied water for watermelon production where *L. siceraria* rootstocks are utilized.

## 1. Introduction

Watermelon [*Citrullus lanatus* (Thun.) Matsum. and Nakai] is the most significant globally produced cucurbit crop, with a production of 101 million Mg from 3.05 million ha estimated in 2020 [1]. Currently, the leading watermelon-producing countries are China, Turkey, and Iran, while Brazil is the primary producer in South America, with 2.2 Mg from 98.205 ha [1]. In Chile, watermelon is cultivated only in a reduced area of 2.962 ha [1] compared to South American countries, even though the local demand for the fruit is very high. The crop is grown for its red-fleshed and nutritious fruit, which is rich in natural sugars (e.g., glucose, fructose, and sucrose), lycopene, organic acids (e.g., malic, citric, and oxalic acids), amino acids (e.g., Citrulline and arginine), essential nutrients (e.g., N, P, K, Ca, Mg, Fe and Zn) and other phytochemical compounds [2,3,4,5,6,7].

In recent years, drought has become one of the most predominant abiotic stresses affecting watermelon production, especially in arid and semiarid areas [8]. Several studies reported that watermelon reaches the highest yield under full irrigation conditions, and water stress conditions result in significant crop yield reductions [9,10,11,12]. In Central Chile, characterized by a Mediterranean-type climate where the summer season is dry and warm, water availability is critical to ensure sufficient watermelon yield [13]. Moreover, the increase in water demand in the Central Valley area is a consequence of the increased population, coupled with the identification of industrial and agricultural activities and inadequate water distribution [14]. Therefore, the effective use of the limited water is crucial for sustainable agriculture in most Chilean regions.

Research studies demonstrate that grafting can effectively mitigate the adverse effects of environmental stress on watermelon production. Grafting watermelon onto various cucurbit rootstocks is a widely practiced technique to improve fruit yield, quality, and resistance to biotic and abiotic stresses [15,16,17]. Abiotic stress tolerance of grafted plants is facilitated by the modifications in root traits, such as deeper and more extensive root systems, higher root hydraulic conductance, and faster induction of hormone accumulation [18]. The main cucurbit rootstocks include interspecific hybrids (*Cucurbita maxima* × *Cucurbita moschata*), bottle gourd (*Lagenaria siceraria*), and squash (*Cucurbita pepo*), and (*C. ficifolia*) [17,18]. Recently, citron watermelon (*C. lanatus* var citroides) has emerged as a promising rootstock for improving fruit yield and quality of grafted watermelon [9,19,20]. Currently, the interspecific hybrid *C. maxima* × *C. moschata* is the most commonly used commercial rootstock for grafted watermelon [8,9]. However, other rootstocks, such as *L. siceraria*, have gained popularity due to their attributes such as improved protection against fusarium wilt and root-knot nematodes [21,22], positive effects on fruit yield, and quality of grafted watermelon fruits under adverse conditions [16]. Some critical fruit quality traits conferred by grafting include high sugar and lycopene content, intense fruit flesh color, and improved fruit aroma [23,24].

Despite the benefits of using *L. siceraria* rootstocks, their potential to improve fruit yield and the quality of grafted watermelon under water deficit conditions have been poorly tested. Mashilo [25] identified some South African accessions of *L. siceraria* tolerant to drought stress, which can be considered a potential genetic resource for use as rootstocks for producing grafted watermelon or for rootstock breeding programs. In addition, South African accessions of *L. siceraria* genotypes have been subjected to recurrent periods of droughts when cultivated under arid and semiarid conditions, which led to the development of drought-resilient plants due to their adaptation to local conditions and many years of artificial selection by local farmers [25]. Despite the above, the genetic resources of *L. siceraria* remain under-utilized and largely unexplored globally for producing grafted watermelon, especially to improve fruit yield and quality in water-limited environments. Similarly, some local bottle gourd accessions in Chile have been identified with high levels of drought tolerance [26]. However, these genotypes have never been evaluated as potential rootstocks for watermelon production in local production areas.

The water stress tolerance imparted by cucurbit rootstocks on grafted watermelon has been extensively reviewed [9,27]. Specifically, the research on watermelon has focused on the changes in water use efficiency, yield, and quality parameters of grafted plants onto commercial rootstock varieties. However, the information is limited to other accessions with potential as rootstocks in different agroclimatic conditions. Therefore, a recent study on watermelon under deficit irrigation concluded that using the correct rootstock is crucial for obtaining a high fruit yield [9]. In addition, there are challenges in maximizing water use and increasing crop productivity per water volume applied using different rootstock-scion combinations [8,9]. One approach for improving water productivity in different rootstocks is to apply less irrigation than the optimum crop water requirements; a deficit irrigation (DI) strategy.

DI is a common practice throughout the world, especially in dry regions, where it is more critical to maximize crop water productivity rather than the harvest per unit of land [8,28,29]. In this practice, the irrigation schedule for implementing a DI strategy can be estimated using the crop evapotranspiration (ETc) demand. As watermelon grows in the summer, when evapotranspiration demands are highest and rainfall is scarce, identifying the best practices for water management using DI techniques is crucial. For now, some research has implemented different irrigation programs under limited irrigation conditions to evaluate the effect on the yield and quality of watermelon fruits [8,9,30]. The results revealed that the yield values obtained from plants grafted onto *C. lanatus* var citroides rootstock with an applied irrigation level of 75% ETc were higher than plants with an applied 100% ETc irrigation level [9]. Additionally, Seymen [30] evaluated wild watermelon as rootstock and concluded that using this breeding material will contribute to the fruit quality in areas with limited water resources. Despite all the information published and available, there is little available data on DI for watermelon grafted on different rootstock materials, especially under the Mediterranean-type climate of Central Chile. Thus, the objective of this study was to evaluate the fruit quality and yield, root system architecture, and water productivity of watermelon grafted onto *Lagenaria siceraria* rootstock.

## 2. Results

The weather conditions over the experimental field were hot and dry during the trial. Supplementary Table S1 indicates that mean values of air temperature were between 15.4 and 20.8 °C, while those of relative humidity ranged between 56.5 and 56.9%. Furthermore, the daily maximum solar radiation ranged between 25.7 and 29.7 MJ m^−2^ d^−1^. In the case of wind speed, the extreme values were 1.3 and 5.9 m s^−1^. Meanwhile, the daily ETo ranged between 3.4 and 7.2 mm d^−1^.

### 2.1. Analysis of Variance for Yield Components and Fruit Quality Traits of Watermelon cv. Santa Amelia F1 Grafted onto L. siceraria Rootstocks

Analysis of variance showed a significant effect of the rootstocks for number of fruits per plant (NFP). On the contrary, for the fruit quality traits of fruit width (FW), fruit rind thickness (FRT), soluble solid content (SSC), luminosity (L), chroma (C), and hue (°h), there were no statistically significant differences among rootstocks, except for fruit length (FL) and L (Table 1). The interaction effect (R*I) showed a statistically significant effect only for total fruit weight, luminosity, and water productivities (WPM and WPT) (Table 1). The rootstock ‘Osorno’ showed statistical differences compared to other rootstocks (except for ‘GC’) regarding the number of fruits per plant (NFP) for grafted watermelon. However, ‘Osorno’ showed significantly higher fruit length than ‘GC’ (Table 2).

### 2.2. Water Productivity of L. siceraria Rootstocks

**Figure 1:**
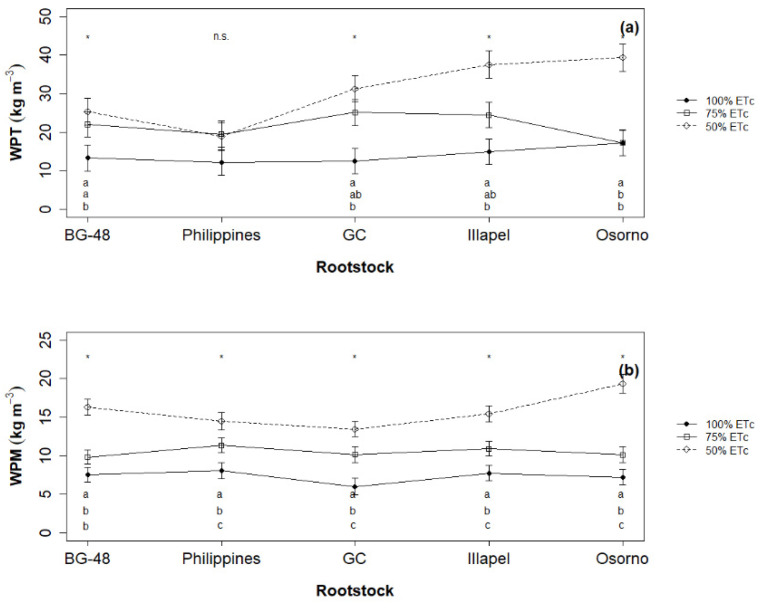
Water productivity based on total yield (WPT, kg m^−3^) (**a**) and marketable yield (WPM kg m^−3^) (**b**) at three irrigation levels of watermelon cv. Santa Amelia F1 grafted onto five *L. siceraria* rootstocks. * and n.s denote the level of significance at 5% and non-significant re-spectively, according to Fisher’s LSD test with Bonferroni correction.

**Figure 2:**
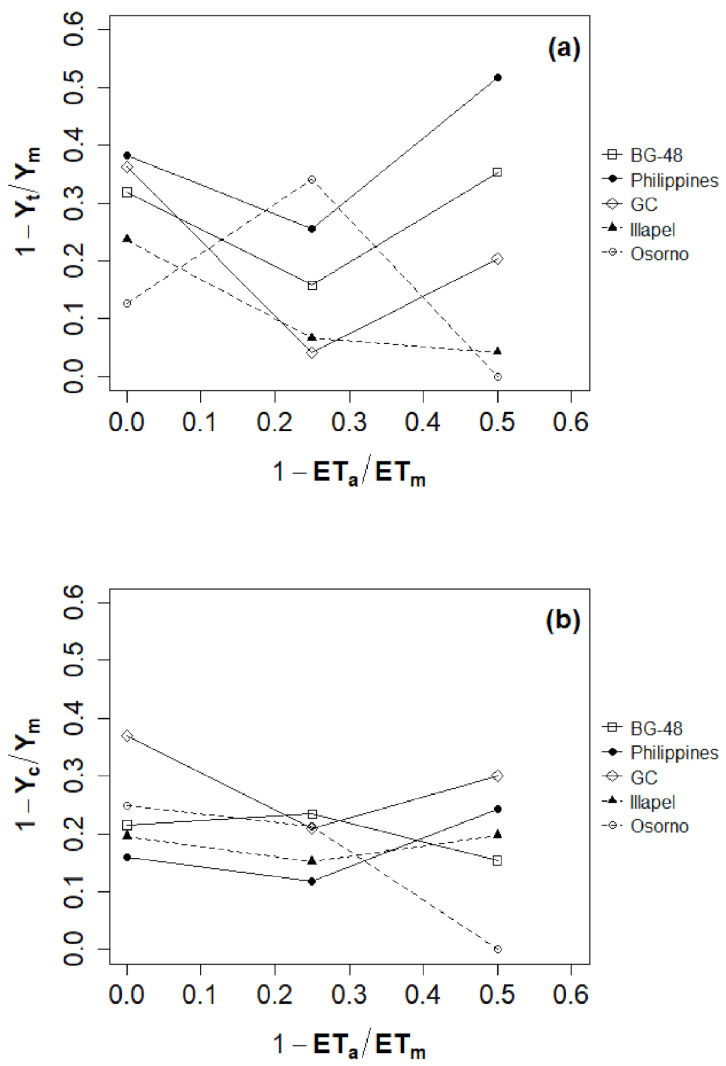
Relationship between relative evapotranspiration deficit (1 − ET_a_/ET_m_) and relative total yield decrease (1 − Y_a_/Y_m_) (**a**) and relative marketable yield decrease (1 − Y_c_/Y_m_) (**b**) of watermelon cv. Santa Amelia F1 grafted onto five *L. siceraria* rootstocks.

Total water productivity (WPT) measured at IR3 ranged from 18.9 kg m^−3^ for ‘Philippines’ to 39.3 kg m^−3^ for ‘Osorno’ (Figure 1a), and a significant increase in the values of marketable water productivity (WPM) was achieved with the irrigation level of 50% (IR3 = 30.4 ± 8.6) (Figure 1b), which was double that of the irrigation level of 100% (IR1 = 14.0 ± 2.1) (Supplementary Table S2). The yield response factor ($k_{y}$) was calculated for each rootstock based on total yield (Figure 2a) and marketable yield (Figure 2b). The $k_{y}$ values indicate the level of tolerance of a crop to water deficit, where the greater $k_{y}$ values represent the lower tolerance. In our results, the $k_{y}$ value was higher in ‘GC’ (1.17) compared to ‘Osorno’ (0.61). This result reveals a greater tolerance of ‘Osorno’ accession to water shortage.

### 2.3. Analysis of Variance for Root System Architecture Traits of L. siceraria Rootstocks Grafted onto Watermelon cv. Santa Amelia F1

**Figure 3:**
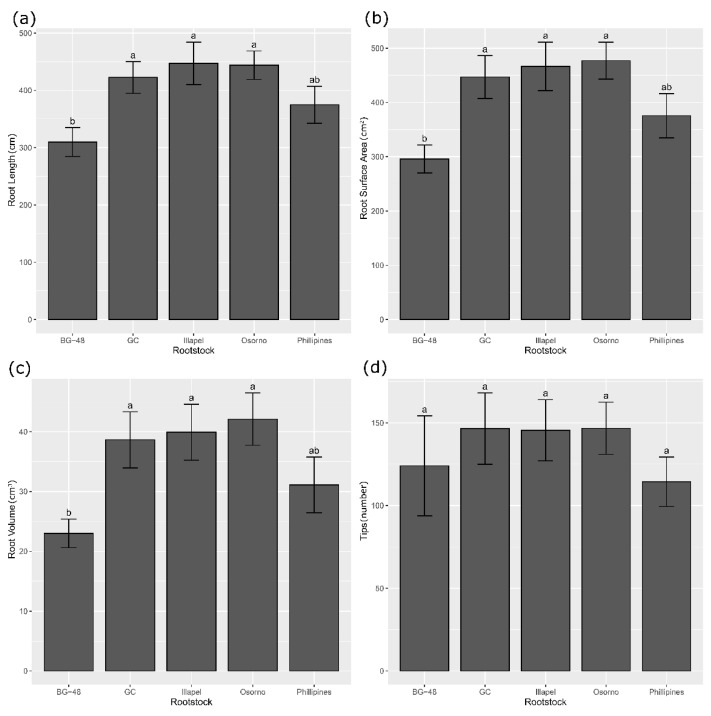
Root system architecture traits of *L. siceraria* rootstocks grafted onto watermelon cv. Santa Amelia F1 and grown under field conditions. (**a**) Root length, (**b**) surface area, (**c**) root volume, and (**d**) number of tips. Different letters indicate significant differences according to Fisher’s LSD test (*p* ≤ 0.01) with Bonferroni correction.

The ANOVA showed statistical differences among the rootstocks for root traits (TRL, SA, RV, and NT). Contrastingly, the irrigation level and interaction effect were not statistically significant in all the root traits (Table 3). Significant differences among the rootstocks were observed for root traits. ‘Osorno’, ‘Illapel’, and ‘GC’ accessions were statistically different from ‘BG-48’ and ‘Philippines’ for most of the root’s traits, except for the number of tips. These accessions showed the highest values for root length, surface area, and volume (Figure 3).

### 2.4. Correlation Analysis of Fruit Yield Components and Quality Traits

**Figure 4:**
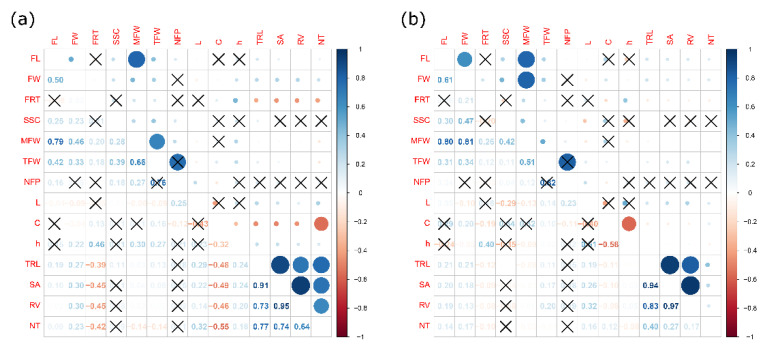
Plot representing the Pearson correlation coefficients among yield (MFW, TFW and NFP), fruit quality (FL, FW, FRT, SST, L, C, and h), and root traits (total root length: TRL, root volume: RV, number of tips: NT, and surface area: SA) of *L. siceraria* rootstocks grafted onto watermelon cv. Santa Amelia F1 and evaluated at the irrigation level of 100%-IR1 (**a**) and 30%-IR3 (**b**). Positive correlations are displayed in blue and negative correlations in red. The color intensity and the size of the circle are proportional to the correlation coefficients. On the right side of the correlogram, the color legend shows the correlation coefficients and the corresponding colors. The intensity of each coefficient of correlation denotes the level of significance, whereas the cross represents no significant correlation between traits.

The Pearson correlation coefficients among WP, yield components, fruit quality, and root traits at the irrigation level of 100% (4A) and 50% (4B) are presented in Figure 4. At both irrigation levels (100% or IR1 and 50% or IR3), a positive and significant correlation was observed among all the root traits. The total fruit weight (TFW) showed a moderate correlation with the marketable fruit weight (MFW) (0.66 at IR1 and 0.51 at IR3). Additionally, MFW positively correlated with fruit length (0.79 at IR1 and 0.80 at IR3) and fruit width (0.46 at IR1 and 0.81 at IR3). The root traits were not correlated with yield components and fruit quality traits at both irrigation levels. Similarly, at both irrigation levels, the color parameters (L, C, and °h) did not significantly correlate with fruit length and width.

### 2.5. Principal Component Analysis

**Figure 5:**
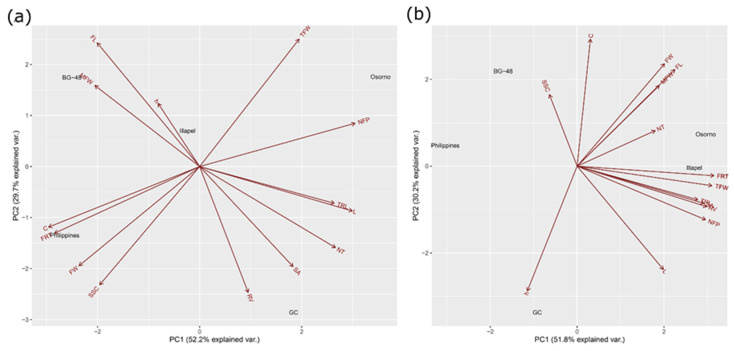
Principal component analysis (PCA) biplot showing variation explained for fruit yield and quality traits, water productivity and root system architecture of *L. siceraria* rootstocks grafted onto watermelon cv. Santa Amelia F1 and evaluated at the irrigation level of 100%-IR1 (**a**) and 50%-IR3 (**b**).

Under the IR1 condition, the two first principal components (PCs) explained 88% of the cumulative variance observed, with 50% at the first and 33% at the second axes (Figure 5a). The parameters that contributed to positive loading to the first principal component (PC1) were TFW, NFP, L, TRL, NT, SA, and RV; while for the second component (PC2), the parameters were MFW, FL, h, TFW, and NFP. In contrast, the parameters °h, C, FL, FW, MFW, FRT, and SSC contributed to negative loading to the PC1, while C, L, FRT, FW, SSC, RV, SA, NT, and TRL contributed with negative loading to the PC2.

Under the IR3 condition, the first two PCs explained 78% (51 and 27%, respectively). PC1 was composed mainly of positive loadings (C, L, FW, FL, FRT, MFW, NFP, TFW, TRL, SA and RV) and only two negative loadings (C and °h). In contrast, PC2 consisted of FRT, TFW, TRL, SA, RV, NFP, L, and °h as negative loadings, and SSC, C, FW, FL, MFW, and NT as positive loadings (Figure 5b).

At the irrigation level of 50% of ETc, the rootstocks ‘Osorno’ and ‘Illapel’ were grouped with high values of root (TRL, NT, SA, and RV), fruit quality (L, FW, FL, and FRT), and some of the yield parameters (TFW and NFP). On the other hand, ‘BG-48’, ‘GC’, and ‘Philippines’ were grouped independently (Figure 5b).

## 3. Discussion

Drought stress is one of the most important abiotic factors restricting watermelon production, causing a significant decline in fruit yield and quality [8,9,31]. Grafting watermelon onto various cucurbit rootstocks has been demonstrated to be an effective strategy to improve fruit quality, yield, and resistance to abiotic stress predominant in semiarid environments [8,9,30]. In this context, we compared different rootstocks as breeding materials to evaluate watermelon’s fruit yield and quality under water deficit conditions.

In the current climate change scenario, to save water and contribute to the fruit quality, different species, as well as *C. lanatus* var citroides (citron watermelon), *L. siceraria* (bottle gourd), and *C. maxima* × *C. moschata* (cucurbit hybrid) have been evaluated as rootstocks under arid and semiarid conditions [8,9,30]. Moreover, rootstocks have been demonstrated to reduce the effect of water stress without affecting the quality and, to a lesser extent, the yield of watermelon fruits [9,30]. In the present study, the grafted watermelon plants (Royal Sweet variety) onto rootstocks of bottle gourd of Chilean origin (Illapel and Osorno) improved the fruit number and yield (total fruit weight) under the irrigation of 50% of ETc (Supplementary Table S3). Those results are consistent with the findings of Khankahdani [32] and Yetisir and Sari [15], who found that grafting watermelon (Crimson Sweet and Crimson Tide variety, respectively) onto bottle gourd rootstock enhances production by increasing both mean fruit mass and number. Contrary to our findings, Yavuz [9] reported that watermelon plants grafted onto bottle gourd rootstocks did not affect the yield under arid and semiarid climatic conditions. This discrepancy may be attributed to agroclimatic conditions, cultivation techniques, particular rootstock–scion compatibility, or because they compared with non-grafted watermelon plants. Another reason for this contradiction was the different watermelon cultivars used in these studies. Despite these differences, our results indicated that the *L. siceraria* evaluated here could be used as commercial watermelon rootstocks to improve the resistance of grafted watermelon plants to drought stress.

High-quality watermelon fruits are required in the marketplace by consumers. The fruit quality of watermelon fruit is often determined by a combination of sweetness, aroma, acidity, chewiness, flesh firmness, and juiciness [33]. In general, water deficit reduces the yield of vegetable crops but, in many cases, improves their quality [31]. In watermelon, for example, the use of rootstocks contributed significantly to the fruit quality under water-limited conditions [8,30,31]. In the present study, *L. siceraria* rootstocks did not affect watermelon fruit quality at 50% of ETc. According to the standards for watermelon fruit quality, the SSC must be equal to or greater than 8° Brix, with fruits similar in shape and color [33,34]. Our results indicate that under water deficit conditions, the SSC values for each rootstock–scion combination were, on average, 10 °Brix, with fruits of the same shape and color (data not shown).

Water productivity is an important indicator of water–yield relations expressed as a unit of water consumption rate [9]. It is also a measure of drought tolerance. In this sense, to enhance agriculture’s sustainability, it is necessary to develop genotypes with high yields and water productivity [26]. A review that summarizes the results of crop water productivity values for irrigated watermelon from seventy-seven studies showed that the range of WP was extensive, ranging from 2.70 to 14.33 kg m^−3^ [35]. In our study, the marketable WP values for watermelon grafted on the *L. siceraria* rootstocks ranged from 13.5 kg m^−3^ for ‘GC’ to 19.3 kg m^−3^ for ‘Osorno’ in water deficit conditions, and these values were higher than the values recorded at the irrigation level of 100% of ETc, and slightly similar when compared with the published for grafted watermelon plants. For example, in watermelon grafted on *C. moschata* × *C. maxima* hybrid, the WP values ranged between 13.2 and 14.5 kg m^−3^, with higher values obtained in water deficit conditions [31]. In addition, similar values of WP were reported by Yavuz [9], with values ranging between 12.1 (for Citron watermelon rootstock) and 15.4 kg m^−3^ (for the hybrid rootstock-TZ148, *C. maxima* × *C. moschata*). The variability range of WP values depends on the experimental year and treatments (i.e., water deficit condition, nutrient application, and so on); moreover, other research has reported a higher WP value equal to 35.6 kg m^−3^ for watermelon grown under greenhouse conditions in the Campo de Dalías, Spain [36]. Despite the WP values reported for watermelon, our results indicate that bottle gourd rootstocks significantly increase the WP at the irrigation of 50% of ETc compared to the irrigation at 100% of ETc. In this sense, these rootstocks could help growers to save water in watermelon production under water deficit conditions in Chile. Therefore, if the watermelon is grafted onto Chilean bottle gourd rootstocks and irrigation water is applied at 50% of ETc, considerable savings in irrigation water could be achieved in the cultivated areas of the Central Region of Chile.

Another important indicator for evaluating different irrigation programs implemented under water deficit conditions corresponds to the yield response factor ($k_{y}$), which expresses the relationship between the decrease in water consumption and the decrease in yield [9]. Based on this parameter, some previous studies indicate that watermelon was quite sensitive to soil water deficit during the total growing period (according to the $k_{y}$ value of 1.1 and 1.07) [37,38]. In addition, Erdem and Yuksel [39] concluded that plants should not undergo a water deficit during the growing season to obtain a high fruit yield. Furthermore, the study of Doorenbos and Kassam [37] stated that if $k_{y}$ > 1, the plant is sensitive to water scarcity. In our study, the $k_{y}$ values calculated with marketable watermelon fruits were from 0.61 for the grafted plant with ‘Osorno’ to 1.17 for ‘GC’ rootstock, indicating that ‘Osorno’ was tolerant and ‘GC’ sensitive to water deficit. The accession ‘GC’ only produced one marketable fruit per plant; consequently, its yield response factor was affected. However, when the $k_{y}$ values were calculated with total cumulative fruits (marketable and non-marketable fruits), significant differences were observed in the yield response factor of the grafted watermelon plants with the different rootstocks; in this case, the susceptible accessions were ‘BG-48’ ($k_{y}$ = 1.5) and ‘Philippines’ ($k_{y}$ = 1.1), whereas the accessions ‘GC’, ‘Osorno’ and ‘Illapel’ were considered tolerant to water deficit. These results help to support the idea that Chilean bottle gourd accessions are considered tolerant to water deficit based on the response of root traits and water uptake [26].

A well-developed rootstock root system can enhance fruit yield and quality and improve the water-use efficiency of grafted watermelon. The *L. siceraria* rootstock showed varied effects on RSA traits when grafted onto watermelon plants. In addition, PCA analysis identified groups of genotypes with different strategies to tolerate drought stress based on the RSA traits studied; one group was composed of Chilean accessions (Osorno and Illapel), and another corresponded to the ‘GC’ genotype. In our previous study, some non-grafted Chilean bottle gourd accessions (Illapel and Chepica) were grouped based on high values for water productivity, root diameter, and volume and separated from the Asiatic and South African genotypes (GC and BG-58) and ‘Osorno’. When ‘Osorno’ is used as rootstock, this modifies its performance to water deficit conditions. Mashilo [25], who performed a biplot analysis based on the physiological parameters in *L. siceraria* under arid and semiarid conditions in sub-Saharan Africa, suggested ‘GC’ as a drought-tolerant genotype based on high values of physiological traits. Consequently, the present study confirmed the tolerance of this accession when used as rootstocks.

Our preliminary results supported the idea that fine roots are responsible for water uptake and productivity in Chilean bottle gourd accessions under water deficit conditions [26]. In the present study, the Chilean bottle gourd accessions (Illapel and Osorno) and ‘GC’ were classified as tolerant when used as rootstocks and consistently showed high values for root architectural traits (volume, length, and surface area) compared to ‘BG-48’ and ‘Philippines’, which are susceptible to water deficit. Consistently, the rootstocks with more vigorous root traits (Osorno, Illapel, and GC) showed the best performance for yield parameters. In this sense, it is stated that grafting directly affects plant yield [9,40] by interactions of some or all of the following processes: increase of water and nutrient uptake resulting from the vigorous root system of the rootstock [17,41], enhanced production of endogenous hormones [42,43] or improved of scion vigor. Considering this information, probably the separated or joint action of some or all these processes could explain the higher yield observed in watermelon grafted on Chilean (Osorno and Illapel) and South African rootstock ‘GC’ in the current study. However, to confirm this idea, it is necessary to elucidate the phytohormone profiles of watermelon grafted onto bottle gourd rootstock in response to water stress.

## 4. Materials and Methods

### 4.1. Experimental Area

The study was conducted during the 2021–2022 growing season at the experimental field of Angel Reyes Valerio company in the Cachapoal Valley (34°39′ S, 70°88′ W, 570 m above sea level), O’Higgins Region, Chile.

The climate is classified as Mediterranean, with hot and dry summers, and a long dry season (seven to eight months), presenting an annual thermal amplitude greater than 10 °C. The annual average precipitation varies between 300 and 400 mm, mainly concentrated between Autumn and Winter [44].

The soil was classified as Corcolén series (coarse–loamy, mixed, thermal Typic Haploxerolls) with a clay loam texture. The bulk density, and the volumetric soil water content at field capacity and wilting point at the effective rooting depth (40 cm) were 1.39 g cm^−3^, 0.27 and 0.13 m^3^ m^−3^, respectively (Supplementary Figure S1). Soil fertility analysis showed an average pH of 6.7, an 1.8% organic matter content, an electric conductivity of 0.09 dS m^−1^, an interchangeable Ca and Mg of 10.84 cmol kg^−1^ and 1.96 cmol kg^−1^, respectively, and a phosphorus-Olsen and potassium (ammonium acetate extract) concentration of 24 and 204 mg kg^−1^, respectively.

### 4.2. Plant Material and Agricultural Practices

Sweet watermelon cv. Santa Amelia F1 (Seminis^®,^), which is a diploid seeded Royal Sweet variety, was grafted onto five accessions of *L. siceraria*. The five bottle gourd accessions used as rootstocks corresponded to Illapel, Osorno, GC, BG-48, and Philippines, which represent three different geographical origins: South Africa (GC and BG-48), Asia (Philippines), and Chile (Illapel and Osorno) [45].

The incorporation of nutrients was performed by fertigation, following the recommendation described by Crawford and Abarca [46], with minor modifications. Briefly, one week before transplanting the seedlings, 185 kg ha^−1^ (monoammonium phosphate) fertilizer was applied and mixed into the soil using a rotary tiller. At the flowering stage, an additional 185 kg ha^−1^ and 370 kg ha^−1^ of monoammonium phosphate and potassium nitrate were applied, respectively. Additionally, 370 kg ha^−1^ of potassium and ammonium nitrate was applied during the fruit development stage with a drip irrigation system.

### 4.3. Experimental Design and Growing Conditions

The field experiment was conducted in a randomized complete block design (RCBD) in a split-plot scheme of treatments that included two factors: rootstock with five levels (Illapel, Osorno, GC, BG-48, and Philippines) and irrigation strategy with three levels with respect to crop evapotranspiration (IR1: 100%, IR2: 75% and IR3: 50%), totalizing 15 treatments. The irrigation strategy represented the main plot, which was replicated three times, with each replication consisting of a bed (10 m^2^) in which the sub-plot represented the five rootstocks randomized within the main plot. The grafted plants were transplanted when they reached the two-leaf stage into an open field at a spacing of 2.0 m by 3.0 m apart in plastic mulched rows, following traditional practices for grafted watermelon crops [46]. The plots were 10 m long with eight plants in each plot, and the width of the raised bed covered by the plastic mulch was approximately 1.0 m. The external plots were surrounded by similar plots to eliminate border effects.

### 4.4. Water Requirements and Irrigation Treatments

The crop water consumption was based on crop evapotranspiration (ETc), and it was estimated following the single crop coefficient methodology (Equation (1)) according to Allen [47]. Then, historical reference evapotranspiration (ETo) data was used daily with kc values for the different growth stages [47]. The watermelon crop was drip-irrigated with double-line using emitters spaced by 20 cm and a flow rate of 1.4 L h^−1^.

ETc = ETo kc(1)

All treatments were fully irrigated according to their water needs from transplanting to the beginning of flowering. Once the crop reached flowering, the irrigation scheduling was differentiated until the harvest. Three irrigation levels were evaluated, and they corresponded to 100% (IR1), 75% (IR2), and 50% (IR3) with respect to ETc.

To evaluate water use–yield relationships for every rootstock, the yield response factor ($k_{y}$) was computed using evapotranspiration and yield data according to Stewart et al. (1977):

(2) $$\left(1-\frac{Y_{a}}{Y_{m}}\right)=k_{y}\left(1-\frac{ET_{a}}{ET_{m}}\right)$$

where *Y_a_* is the actual harvested yield for every rootstock–irrigation treatment combination, *Y_m_* is the maximum harvested yield, $\;k_{y}$ is the yield response factor, *ET_a_* is the actual evapotranspiration for every rootstock–irrigation treatment combination, and *ET_m_* is the maximum evapotranspiration. Furthermore, the water productivity was computed as a global value for every rootstock, as the ratio of yield to water applied [48].

### 4.5. Yield and Fruit Quality Parameters

Grafted watermelon seedlings were planted on 25 October 2021, and the fruits were harvested on 18 January 2022. Four representative plants were sampled from each plot at the end of the growth cycle (86 days after transplanting). The yield parameters were expressed as the total cumulative fruits, separated into marketable and non-marketable fruits from the sampled plants of each plot. Commercial fruits were classified following the standard classification in Chile; based on the weight and size, fruits with a weight lower than 5 kg are small or non-marketable, and fruits with a weight greater than 7 kg are marketable. The average marketable fruits (MFW) and non-marketable fruits were weighted with a precision balance (Radwag WTC-2000) and the number of fruits per plant (NFP) were counted. The commercial and non-marketable fruit weight was considered as the total fruit weight (TFW).

Fruit quality parameters were measured in four representative commercial watermelon fruits per plot. The selected commercial fruits were cut and measured to determine the fruit length (FL) and fruit width (FW). In addition, the same fruits were used to determine the fruit rind thickness (FRT), and soluble solid content (SSC; °Brix), which was assessed with the juice obtained from the central part of the fruits by using a thermo-compensated refractometer (Proconsa DR301). Flesh color coordinates (L, a, and b) were obtained from the central part of the commercial fruits using a portable colorimeter (Minolta CR-400, Tokyo, Japan) using the CIELab system. L* represents the luminosity, whereas the values of a and b were used to calculate the Hue (°h) and Chroma (C) according to McGuire (1992).

### 4.6. Root Measurements

Three of the four representative plants were sampled from each plot at the end of the growth cycle for root extraction using the shovelomics protocol [49]. The whole root system of each replication of the rootstock–irrigation level treatment was analyzed according to Contreras-Soto [26]. Briefly, the roots were cleaned by washing them over a sieve. Each sample was scanned with a flatbed scanner (Epson Perfection V800 Photo & V850 Pro, Seiko Epson Corp., Japan-resolution 6400 dpi). The total root length (TRL), root surface area (SA), root volume (RV), and the number of tips (NT) were determined by WinRhizo 2019a software (Regent Instruments Inc., Quebec City, QC, Canada).

### 4.7. Statistical Data Analysis

Analysis of variance (ANOVA) was performed after testing the homogeneity of variances and normality of the residuals using Bartlett and Shapiro–Wilk tests, respectively. A mixed linear model was performed for yield components, fruit quality, and RSA traits with the PROC MIXED procedure of SAS software [50]. Fisher’s least significant difference (LSD) with Bonferroni correction was performed for multiple comparisons of mean values of rootstocks or irrigation level. For the variable number of fruits per plant (NF), a generalized linear mixed model with multinomial distribution and cumlogit link function was performed using the PROC GLIMMIX procedure of SAS software [50].

The mean values per plot of the studied traits were used to compute Pearson’s linear correlation coefficients using the “corrplot.mixed” function of the corrplot package [51] in R 4.0.5 [52]. A principal component analysis was carried out to identify the contributing traits (yield and quality fruit parameters and root traits) under two irrigation levels (IR1 and IR3), aiming to differentiate drought-tolerant and sensitive bottle gourd rootstocks. PCA and biplot graphics were performed using the “princomp” and “ggbiplot” functions in R 4.0.5 [52] according to Contreras-Soto [26].

## 5. Conclusions

The present study evaluated fruit yield, quality, and water productivity of watermelon grafted onto *Lagenaria siceraria* rootstock to select suitable rootstock–scion combinations for recommendation and cultivation under water deficit conditions. In addition, the root system architecture of *L. siceraria* rootstock when grafted to watermelon was compared with them. Fruit quality of the grafted watermelon was not affected by the used *L. siceraria* rootstocks under water deficit conditions, whereas the rootstocks of ‘Illapel’, ‘Osorno’ and ‘GC’ improved total fruit number and yield and water productivity under water deficit conditions. The rootstocks of ‘Illapel’, ‘Osorno’, and ‘GC’ showed increased root volume, length, and surface area compared to the other rootstocks under water deficit conditions due to their drought-tolerant nature. The present results suggest that *L. siceraria* rootstocks could improve fruit yield and quality of grafted watermelon where its production is under arid, and semiarid environments.

## Figures and Tables

**Table 1:** Type III analysis of rootstocks (R), irrigation levels (I), and their interaction (R*I) on the number of fruits per plant (NFP), marketable fruit weight (MFW), total fruit weight (TFW), fruit length (FL), fruit width (FW), fruit rind thickness (FRT), solid soluble content (SSC), luminosity (L), chroma (C), hue (°h), marketable water productivity (WPM) and total yield water productivity (WPT) of watermelon cv. Santa Amelia F1 grafted into five *L. siceraria* rootstocks under field conditions.

SV	NFP (Number)	MFW (kg)	TFW (kg)	FL (cm)	FW (cm)	FRT (cm)	SSC (°Brix)	L	C	°h	WPM (kg m^−3^)	WPT (kg m^−3^)
R	***	ns	***	**	ns	ns	ns	***	ns	ns	*	***
I	ns	ns	ns	ns	ns	ns	ns	ns	ns	ns	**	ns
R*I	ns	ns	*	ns	ns	ns	ns	*	ns	ns	*	***

SV: source of variation. The levels of significance (ns: non-significant; * significant at 5%; ** significant at 1%; *** significant at 0.1% using the F-test) are indicated. Means followed by different letters are significantly different according to Fisher’s LSD test (*p* ≤ 0.01) with Bonferroni correction.

**Table 2:** Effect of five *L. siceraria* rootstocks on the number of fruits per plant (NFP) and fruit length (FL) of grafted watermelon cv. Santa Amelia F1 grown under field conditions.

Rootstocks	NFP (Number)	FL (cm)
Osorno	2.37 a	35.58 a
Illapel	2.13 bc	35.37 a
GC	2.31 a	32.58 b
Philippines	1.45 c	35.00 ab
BG-48	1.69 bc	35.25 a

Means followed by different letters are significantly different according to Fisher’s LSD test (*p* ≤ 0.01) with Bonferroni correction.

**Table 3:** Type III analysis of rootstocks (R), irrigation levels (I), and their interaction (R*I) on the total root length (TRL), root surface area (SA), root volume (RV), and the number of tips (NT) of five *L. siceraria* rootstocks grafted onto watermelon cv. Santa Amelia F1 and grown under field conditions.

SV	TRL (cm)	SA (cm^2^)	RV (cm^3^)	NT (Number)
R	***	***	***	**
I	ns	ns	ns	ns
R*I	ns	ns	ns	ns

SV: source of variation. The levels of significance (ns: non-significant; ** significant at 1%; *** significant at 0.1% using the F-test) are indicated. Means followed by different letters are significantly different according to Fisher’s LSD test (*p* ≤ 0.01) with Bonferroni correction.

## Data Availability

The data presented in this study are available upon request from the corresponding author.

## Supplementary Materials

The following supporting information can be downloaded at: https://www.mdpi.com/article/10.3390/plants12030509/s1, Table S1: Summary data of the meteorological conditions during the trial characterized by the air temperature (Ta), relative humidity (RH), daily solar radiation (Sr), wind speed (Ws), and reference evapotranspiration (ETo); Table S2: Mean values from the analysis of variance for the total water productivity (WPT) and the marketable water productivity (WPC) for every combination of rootstock and irrigation level (100%, 75% and 50% ETc); Table S3: Mean values from the analysis of variance for the total fruit weight (TFW) for every combination of rootstock and irrigation level; Figure S1: Volumetric water content measured at 20 cm (a) and 40 cm (b) of the soil each week during the experiment.
