# Supplementary material for: Rootstocks Comparison in Grafted Watermelon under Water Deficit: Effects on the Fruit Quality and Yield
Source: Plants (Basel). 2023 Jan 22;12(3):509. doi: 10.3390/plants12030509 (PMC9920548; doi:10.3390/plants12030509)

Table S1. Summary data of the meteorological conditions during the trial characterized by the air temperature (Ta), relative humidity (RH), daily solar radiation (Sr), wind speed (Ws), and reference evapotranspiration (ETo).

	**Ta (°C)**	**RH (%)**	**Sr (MJ m^{-2} d^{-1})**	**Ws (m s^{-1})**	**ETo (mm d^{-1})**
October ^{S2021}					
Min	-0.1	13.7	6.0	1.3	1.3
Max	30.8	99.9	25.7	6.8	5.2
Avg	15.4	56.8	15.9	4.1	3.2
November ^{S2021}					
Min	4.2	13.4	15.7	2.0	2.7
Max	32.1	100.0	27.9	8.7	5.9
Avg	18.2	56.7	21.8	5.4	4.3
December ^{S2021}					
Min	6.9	13.8	7.0	2.2	1.6
Max	34.7	100.0	29.7	4.4	5.8
Avg	20.8	56.9	18.4	3.3	3.7
January ^{S2022}					
Min	6.4	15.1	12.0	2.2	2.5
Max	34.1	97.8	28.8	4.5	5.7
Avg	20.3	56.5	20.4	3.4	4.1

S2021 and S2022 correspond to season 2021 and 2022, respectively; Min, Max, and Avg corresponds to the minimum, maximum and average values, respectively.

Table S2. Mean values from the analysis of variance for the total water productivity (WPT) and the marketable water productivity (WPM) for every combination of rootstock and irrigation level (100%, 75% and 50% ETc).

**Rootstock**	**100% ETc**		**75% ETc**		**50% ETc**	
	WPT (kg m^{-3})	WPM (kg m^{-3})	WPT (kg m^{-3})	WPM (kg m^{-3})	WPT (kg m^{-3})	WPM (kg m^{-3})
BG-48	13.3	7.6	22.0	9.9	25.4	16.3
Philippines	12.1	8.1	19.5	11.3	18.9	14.5
GC	12.5	6.0	25.1	10.2	31.3	13.5
Illapel	15.0	7.7	24.4	10.9	37.5	15.4
Osorno	17.2	7.2	17.3	10.1	39.3	19.3

Table S3. Mean values from the analysis of variance for the total fruit weight (TFW) for every combination of rootstock and irrigation level.

Rootstock	Irrigation (ETc)	TFW (Kg)
Osorno	50	22.94 a
GC	75	21.99 a
Illapel	50	21.97 ab
Illapel	75	21.42 abc
Osorno	100	20.05 abc
Philippines	75	19.31 abc
GC	50	18.28 abcd
Illapel	100	17.51 abcd
BG-48	75	17.09 abcd
Philippines	100	15.62 abcd
Osorno	75	15.13 abcd
Philippines	50	14.83 abcd
GC	100	14.63 bcd
BG-48	100	14.18 cd
BG-48	50	10.87 d

Figure S1. Volumetric water content measured at 20 cm (a) and 40 cm (b) of the soil each week during the experiment.

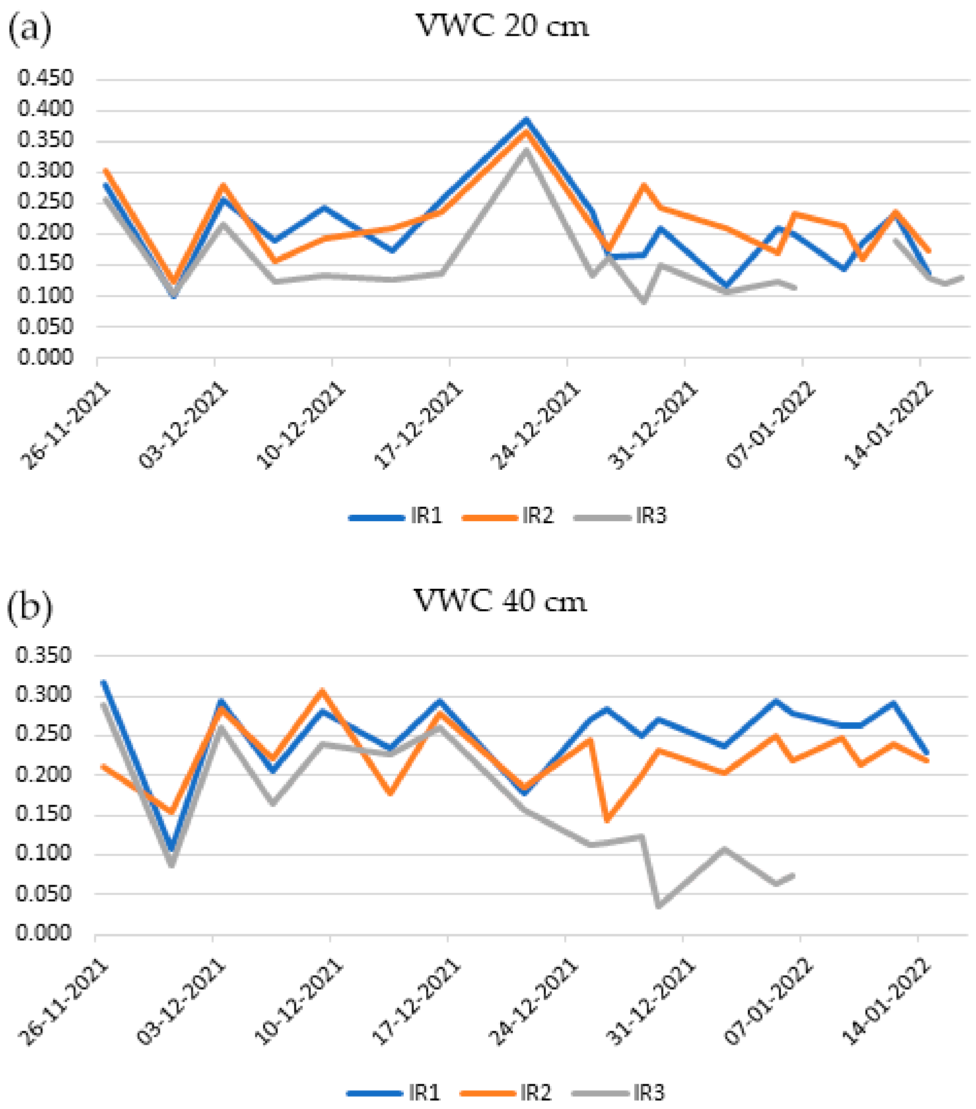

Supplement: Supplementary file 1 [file plants-12-00509-s001.zip › plants-2058425-supplementary.pdf]
